# Working with Parry sound area local Canadian First Nations to describe a good death and ensure cultural sensitivity at the end-of-life

**DOI:** 10.1186/s12904-025-01880-6

**Published:** 2025-09-29

**Authors:** C-M Latcu, L Allen, N Forfar, M Partyka-Sitnik, D Pegahmagabow, C Tryon, J Smith-Turchyn, J.L Davis

**Affiliations:** 1 Family Medicine, Northern Ontario School of Medicine University, Sudbury, ON Canada; 2 Parry Sound Local Education Group, Parry Sound, ON, Canada; 3 Shawanaga First Nation Healing Centre, ON, Canada; 4 Wasauksing First Nation, ON, Canada; 5 Moose Deer Point First Nation, ON, Canada; 6 School of Rehabilitation Science, McMaster University, Hamilton, ON Canada; 7 West Parry Sound Health Centre, Parry Sound, ON, Canada

**Keywords:** First nations, Good death, Cultural sensitivity, Palliative care, End-of-Life care, Family, Community, Aboriginal, Parry sound, Nothern ontario, Canada

## Abstract

This study explores the culturally sensitive palliative and end-of-life care within First Nations communities in the Parry Sound area: Shawanaga, Wasauksing, and Moose Deer Point First Nations communities in Canada. The legacy of colonialism, particularly the Indian Act of 1876, has significantly disrupted Indigenous health practices, contributing to mistrust in Western healthcare systems. This research addresses gaps in culturally appropriate palliative and end-of-life care by identifying how First Nations communities define a “good death” and how Western healthcare providers can respect and integrate these traditions, particularly in rural and remote communities. Using a community-driven approach, three sharing sessions were conducted with 19 participants, exploring their insights into palliative and end-of-life care. Data analysis revealed five key themes: (1) Family members as primary caregivers, (2) Local healthcare providers support family in palliative and end-of-life care, (3) Make ‘final journey’ at home, (4) Community (the ‘clan family’) is involved in palliative and end-of-life care, and (5) Individual wishes for end-of-life care vary and should be followed by healthcare providers. These findings emphasize the role of family, community, and spiritual beliefs in shaping a “good death.” The study calls for healthcare providers to incorporate cultural sensitivity, support home-based care, and collaborate with community leaders to bridge gaps between Western medicine and Indigenous traditions. Recommendations include fostering trust with healthcare providers, ensuring care aligns with cultural values, and enhancing collaboration between healthcare systems and Indigenous communities. This research contributes to improving palliative and end-of-life care for First Nations communities by promoting culturally safe, person-centred care practices.

## Introduction

The Indian Act of 1876 initiated a legacy of forced assimilation for First Nations, Métis, and Inuit people in Canada, with profound and enduring impacts on their cultural, spiritual, and healthcare practices [1]. Among its many harms, the act institutionalized residential schools and segregated healthcare facilities that disrupted traditional ways of life, particularly practices surrounding health, death, and dying [2, 3]. This systemic oppression led to a significant loss of cultural knowledge, ceremonies, and healing practices, creating barriers to grief processing and family healing [2, 4]. Today, these historical inequities contribute to ongoing mistrust of Western healthcare systems, particularly in palliative and end-of-life (PEOL) care [5]. 

For Indigenous people, life and death are seen through a holistic lens, emphasizing the continuous circle that passes the spirit from one generation to the next in death ceremonies, as opposed to the linear approach predominant in white populations [2]. Death ceremonies involving traditional attires, practices, and community gatherings are central to this worldview [1, 6]. The healthcare systems in Canada, often focused on individual care within institutional settings, lacks the flexibility to accommodate these practices [1, 4]. This disconnect leaves many Indigenous patients and families feeling isolated and alienated during critical PEOL moments [2, 4, 6]. 

Systemic barriers further complicate access to culturally relevant PEOL care, particularly in rural and remote communities. Indigenous communities face reduced life expectancy due to a disproportionate burden of disease, compounded by limited access to healthcare and socioeconomic inequities [7, 8]. Many Indigenous individuals wish to die at home, surrounded by family and traditions, but a lack of culturally trained providers and appropriate resources (infrastructure, and support systems) prevents this [4–6]. These gaps highlight the urgent need for culturally safe care that respects Indigenous traditions and addresses the mistrust rooted in colonial trauma [1, 5, 8]. 

Despite these challenges, solutions are emerging. Initiatives like Indigenous navigators, telemedicine, and community-based care have shown promise in bridging the gap between Western medicine and traditional practices. Elders involvement in cultural safety and education is also paramount for success [1, 2, 6, 7]. These approaches help create culturally safe spaces for PEOL care by fostering collaboration between healthcare providers and Idigenous communities [6]. However, significant research gaps remain regarding how First Nations, Métis, and Inuit communities define a “good death” and how their needs can be effectively integrated into PEOL care frameworks, particularly in rural and remote areas [8]. Therefore, the overall objective of this study was to explore the culturally sensitive palliative and end-of-life care within three First Nations communities in Canada.

While this study centers on First Nations communities in Northern Ontario, its contribution also aligns with broader literature emphasizing the significance of culturally concordant care at PEOL care. Prior studies across diverse populations have underscored the importance of recognizing spiritual beliefs, family roles, and traditional practices in PEOL care. Integrating such perspectives enhances the cultural safety and relevance of care, while addressing persistent gaps in accessibility and respect for cultural identity at this critical time [9–11]. 

## Materials and methods

### Study design

This was a qualitative descriptive study using sharing circle methodology. Qualitative descriptive studies are used to provide clear description of the explored research question and have theoretical foundations in naturalism [12]. Sharing circles/sessions are much like focus groups, but are a traditional Indigenous practice fostering open, inclusive, and respectful dialogue [12]. Sharing circles promote equitable participation, often guided by Elders or symbolic items, creating a culturally safe space for storytelling and reflection [13]. 

### Participants and recruitment

Participants were invited to participated through community networks guided by Indigenous community leaders and cultural advisors to ensure diverse representation. Participation opportunities were shared through local newsletters and social media. We actively sought continuous input and feedback (invitations email, questions, in-person introductory sessions) from key stakeholders, including the communities’ Chief, the Chief’s counsellor, the executive director and assistant of the Healing Centre, the director of the Health Centre, and the Health Representative/Community Health Coordinator. These trusted leaders, familiar with participants and their cultural contexts, helped facilitate engagement and foster trust. The inclusion criteria for participation were self-identifying First Nations members with experiences or insights related to PEOL care. No exclusion criteria were applied.

### Data collection

Data was collected during in-person meetings at the three Indigenous communities Healing Centres. Each session lasted approximately 2 h and was recorded and transcribed verbatim. The sharing sessions were moderated by a research team member trained in qualitative research methods, with assistance from an assistant and community liaison members. A semi-structured script was used to guide the focus group and included 9 open-ended questions, with several probes for more information or clarification as needed. The questions focused on what a “good death” means to members of these communities and how Western medical providers can support First Nations communities in the provision of PEOL care. The semi-structured script was co-developed with Indigenous community leaders and cultural advisors. Input was sought during preliminary meetings with representatives from each communities’ health leaders. They reviewed the wording, structure, and cultural appropriateness of the questions to ensure alignment with community values and language. A copy of the semi-structured script can be found in Table 1. The assistant made field notes and summarized major discussion points during the session. One-on-one semi-structured interviews were offered to promote confidentiality, but none was requested.

**Table 1:** Sharing sessions semi-structured script

1. In your culture, is it ok to talk about death and dying?
a. Who would you (or would you not) speak to
b. Is this a taboo subject or one to be shared
2. What medical ceremonies or traditions are important in your culture when it comes to taking care of people who are very sick or near the end of their life? Please explain them if you can/are comfortable?
a. Are any of these ceremonies particularly meaningful and/or important before or after death
b. Are any of these time sensitive (need to be completed within a specified time of death)
c. Are there any activities/behaviours that should not be done at the End of Life
3. When a member of your community is sick (palliative), or near their end of life who traditionally takes care of them, and where?
a. Is this a traditional role? What is the meaning of this role for both the caregivers and the patient?
b. Who do you think should help take care of people who are very sick or near the end of their life in your community?
i. *Probe if needed: Elders*,* Senators*,* Chiefs*,* investigators*,* and/or other community members?*
4. In your culture, where do community members who are near end of life or palliative live?
a. Where do you think these patients should/want to receive care (hospital, home, Nurse Practitioner station)?
b. In your experience do patients receive the care they want in the place they want to be? Why or why not?
5. In your own words, what is a ‘good’ death?
a. ls this presently available for you or members of your community?
6. Do you have the resources you need to support patients close to their end of life?
a. Are you supported in your community to die at home?
b. How could you be better supported for meaningful end of life care in your community?
7. How can we/Western Medicine providers/doctors change what we are doing to better support your community?
8. What results or changes in Palliative and End of Life care do you hope to see from our team’s work?
a. How can we support your community and families as they deal with loss of a loved one.
b. What is not currently done in Palliative care that could be done to support your traditions and ways
9. End with: Have we missed asking a question that you believe is important? Do you have anything else to add?

### Data analysis

Qualitative responses were analyzed using Braun and Clarke’s (2006) six-step thematic analysis [14]. Two authors independently analyzed transcript data from each group using NVivo software to inductively identify codes and collaboratively developed a codebook. Transcripts were then analyzed by one reviewer using the developed codes to finalize themes. Researchers performing coding communicated as needed during final coding to discuss any new codes that emerged and address discrepant interpretations. While community liaisons were involved throughout the design and data collection phases, they did not participate directly in data analysis, however, they reviewed analysis findings as a form of member checking. Saturation of data was ensured during the coding process. We used reflexive thematic analysis and embraced reflexive engagement with the data. This process also aligns with Braun and Clarke’s updated guidance on reflexive thematic analysis (2024) [15]. 

## Results

### Participants

**Fig. 1:**
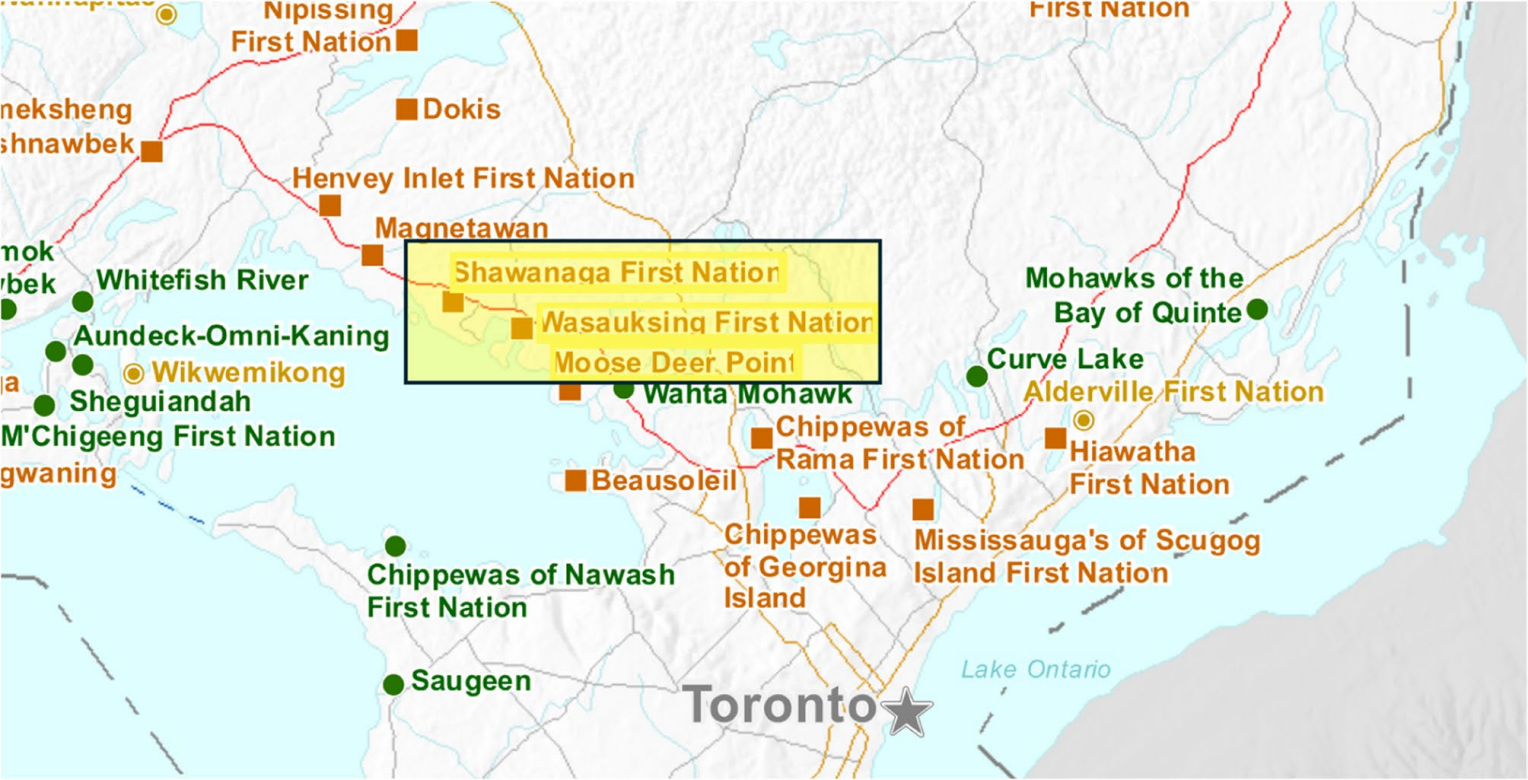
Participating First Nations communities (sites highlighted in yellow)

Between September and October 2024, three sharing sessions were conducted across three First Nations communities in the Parry Sound area: Shawanaga, Wasauksing, and Moose Deer Point, with a total of 19 participants (five participants from Shawanaga, five from Wasauksing, and nine from Moose Deer Point). Demographic description of the participants and community participation details can be found in Table 2. Figure 1 shows their locations in Ontario.

**Table 2:** First Nation communities characteristics (ref: government of Canada) and participation to the study by community

	First Nations Community		
	Shawanaga First Nation (adult population *n* = 160)	Wasauksing First Nation (adult population *n* = 250)	Moose Deer Point First Nation (adult population *n* = 170)
Total population *N*	195	320	210
Age (median)	37.4 years	40 years	39.4 years
Sex (n(%) male/n(%) female)	95(49%)/100(51%)	160(50%)/155(48%)	100(48%)/105(50%)
Language (% with knowledge of Indigenous language)	10.3%	40.6%	16.7%
Marital status (of total adult population (n (%))*: Married Separated Divorced Widowed Never Married	65 (41%) 15 (9%) 10 (6%) 15 (9%) 55 (34%)	115 (46%) 10 (4%) 15 (6%) 20 (8%) 85 (34%)	105 (62%) 10 (6%) 10 (6%) 20 (12%) 45 (26%)
Education level (of total adult population (n (%))*: No degree, certificate or diploma High school or equivalent Trades/apprenticeship University certificate below bachelor level University degree (bachelor or higher)	60 (38%) 50 (31%) 50 (31%) 10 (6%) 0	60 (X%) 60 (X%) 90 (X%) 10 (X%) 30 (X%)	70 (33%) 60 (29%) 30 (14%) 10 (5%) 10 (5%)
Participation from total of 19 participants	26%	26%	48%
Participation from adult population	3.13%	2.00%	5.29%
Predominant gender	Women	Women	Women
Language barriers	None	None	None

Age values represent the median age of the adult population in each First Nations community, not the median age of study participants

Row 1 refers to total population size (all ages)

Row 2 refers to adult population

*How Indigenous individuals and families define a “good death”*

Analysis of sharing sessions data identified five main themes that define a “good death” for First Nations communities: (1) Family members as primary caregivers, (2) Local healthcare providers support family in PEOL care, (3) Make ‘final journey’ at home, (4) Community (the ‘clan family’) is involved in PEOL care, and (5) Individuals wishes for end-of-life care vary and should be followed by healthcare providers.

1. *Family members as primary caregivers*. Participants felt that providing PEOL care for family members was their ‘duty’, but a difficult process. This came forth in statements such as:

*“My mom never wanted to go to a nursing home. She spoke about that before she even got really sick. I was approached by several community members*,* ‘Have you ever thought of putting her in a home?’ And I said*,* ‘No*,* she’s not going in a home. That’s what I’m for. I’m here to take care of her. She took care of me. She raised me*,* so it’s my turn to take care of her.’ I mean*,* it’s not easy work. It’s hard.”* (Focus Group 1).

*“He didn’t want anybody else to look after him. He didn’t want anybody else. He didn’t want any of the nurses here. He just wanted them [his family]…That was extremely hard as he progressed*,* because he was always in pain and always feeling nauseous.”* (Focus Group 3).

Participants described the benefits they as caregivers received while caring for their family at home.

*“My mom*,* when she was in the hospital*,* because I took care of her for two and a half years. Some people say it’s a hard thing. Well*,* yes*,* in a sense. But I learned a lot by taking care of my mom. I said*,* ‘Jeez*,* mom*,* I can be a nurse without a license.’ Because I knew a lot*,* because I learned a lot just by taking her to her appointments and asking a lot of questions.”* (Focus Group 1).

(2) Local healthcare providers support family in palliative and end-of-life care. While it was clear that the preference was to receive PEOL care at home, the participants highlighted that support from local healthcare workers eased the caregiving role. This was clear in statements like:

*“The palliative care team here is remarkable. The way they handled everything*,* they helped my mother-in-law*,* they were there [at her home] every day. Homecare was there. It was really*,* really incredible.”* (Focus Group 1).

*“[The community care coordinator] is a blessing to us because I know that she made sure I had a commode in my room*,* and she made sure I had bed rails up on the bed*,* things like that. So*,* to have the PSWs here*,* and [the community care coordinator] is wonderful. [She] is a wonderful leader*,* and she makes sure that you have everything that you need.”* (Focus Group 3).

Participants also said that coordination and communication between hospitals, First Nations community and family members enhances end-of-life care. This came forth in statements like:

*“I think the most important aspect of palliative care is communication. So*,* making sure that at the healing center here*,* whoever is in charge of the palliative care*,* keeping the patients and keeping the family informed and contacting the nurse or helping them and bringing supplies or whatever that needs to be done.”* (Focus Group 1).

(3) *Make final journey at home.* Much of the discussion around a ‘good death’, was centred on the idea of individuals being able to die at home. Death was often described as the journey to the next phases of a spiritual life. The need for comfort and peace during this time was emphasized:

*“If I was in the hospital and I knew I was going to be dying*,* I’d want to go home. I wouldn’t want to be in a strange place in a strange bed*,* even though I know the hospital because I’ve been in there. But it’s still a strange place in a strange bed for when I’m going to make my final journey. I would rather be at home in the comfort of my own bed.”* (Focus Group 1).

To allow people to make their final journey at home, participants described the needed education to assist families in caregiving safely.

*“My father-in-law*,* they kept him at home because he didn’t want to die in the hospital. And we went to visit him…[I noticed] my brother-in-law withheld his pain meds. And when I got there*,* he [my father-in-law] was in pain. And he was just like so stiff with pain. And I’m like*,* ‘You need to give him pain meds.’ I had to call the police and the ambulance…he wasn’t giving him the meds…He was afraid he was going to choke. So*,* like it’s easy to say*,* take them home…But…there’s lots of things that could go wrong.”* (Focus Group 1).

Further, participants also discussed the need for resource support at home (such as equipment, healthcare providers, and the local healing centre) to ensure the safety of their loved ones. This came forth in statements like:

*“The equipment [needed]…not all home doors are wide enough [for the wheelchair]. I know this one family had great difficulty every time the ambulance would come and bring their father home because the doorways would not fit them….they could not fit through the door and the angle…’how do you get in?’. Sometimes the resources needed to support that care [at home] are challenging or not there at all*,* like the physicality of it.”* (Focus Group 2).

(4) *Community (the ‘clan family’) is involved in palliative and end-of-life care.* Within the group discussions, the importance of community, or the clan family, was repeatedly described as assisting in the PEOL care of others within First Nations communities.

*“Family isn’t small*,* family is big…the clan families*,* we talk about that kind of stuff [end-of-life care and death].”* (Focus Group 1).

Community members supported others by providing food, caregiving, and by holding wakes or other traditional ceremonies. This was discussed in statements like:

*“The social services staff*,* what we call community workers*,* are the ones who run around and get the food for the family and have meals for the family. We do that all the time. Then*,* if there’s a fire*,* then they’re the ones who are also getting the food for the fire crews. As community members*,* we…really support each other in that process*,* so the individual can stay home*,* be cared for by their family.”* (Focus Group 2).

Participants also emphasized the importance of Elders in PEOL care, describing how they provide spiritual guidance and lead ceremonies.

*“We’ve been pretty lucky here… if someone has passed*,* there’s a send-off ceremony from the [healing room] at the hospital where we’ve been able hold it in that room…but we have also gathered people into their hospital room or the emergency room or wherever*,* and the elder would do the send-off there.”* (Focus Group 1).*“Because I carry the feathers*,* I always do my ceremonies to the people who are dying. And*,* I tell you*,* it is the most beautiful experience…to finally say*,* thank you. I’ve attended -- I don’t know how many people who are passing to the spirit world…the last prayer that I say is the one of thanks. Thank you for sharing your life with us. I think that’s really important.”* (Focus Group 3).

(5) *Individuals wishes for end-of-life care vary and should be followed by healthcare providers.* While the importance of community was described during PEOL care, it was highlighted that individual wishes need to be followed by all healthcare providers. This was described:

*“Yes*,* so respecting the wishes of the family*,* the community*,* so that the ceremonies that traditionally happen can happen.”* (Focus Group 2).

*“So*,* the family will know the wishes of the individual*,* who will inform the community*,* and then the community will come together and take part in these ceremonies for the individual.”* (Focus Group 1).

Participants also described variability, family and community dynamics, which affected the openness of First Nations people in discussing death and dying. It was noted that generational changes were occurring, which increased comfort with discussing PEOL, and that efforts to normalize conversations around death and dying are happening within some First Nations communities. This was described in ways like:

*“I talk to my kids about it because I was never taught anything about it. We weren’t allowed to talk about it. So*,* the stuff that I was not allowed to talk about when I was growing up*,* I talked about all kinds of different things with my kids*,* because they need to know. You don’t like being left in the dark.”* (Focus Group 1).

### Recommendations to improve culturally sensitive practice

When exploring how Western medical providers can support First Nations communities and optimize PEOL care, five main categories emerged: (1) Advocacy needed to maximize care received, (2) Effective communication and shared decision-making vital between patients, families and healthcare providers, (3) Taking time to build trust between community members and healthcare providers, (4) Cultural sensitivity training needed for healthcare providers and students, and (5) Create hospital policies and procedures that respect culture and traditional medical practices to support First Nations communities:

1. * Advocacy needed to maximize care received.* Discussions highlighted the need for advocacy for First Nations communities to receive equitable care and respect for their traditional practices in Western healthcare systems. This came forth in statements like:

*“With the prejudice and going to the hospital…[I want to] make sure that they (Indigenous People) get the same comfort as everybody else. And the ones that want cedar baths [get to have them] in the hospital. So*,* I think it’s very important that we have a say.”* (Focus Group 1).

*“I think that doctors and nurses have to be more understanding. My brother*,* when he passed away*,* when he got really sick*,* he had neuroblastoma. So*,* he was in septic shock and honestly*,* in the beginning*,* we asked if there was a navigator [Indigenous navigator]. And there wasn’t one*,* which shocked me because it was a big hospital*,* and it’s surrounded by First Nations. But…within a week*,* they had brought in a navigator…I think you have to have some defense mechanism with doctors and nurses to protect you…It wasn’t until myself*,* my daughter*,* and my granddaughter*,* we dressed in our traditional skirts…and*,* it wasn’t until they actually [saw us]*,* as they’re walking through*,* they’re going*,* ribbon skirts*,* ribbon skirts*,* and we get to hear that. And*,* they were all beginning to show a true interest in who we were and who he was…the atmosphere changed.”* (Focus Group 3).

Participants also felt that advocacy was needed to engage Elders and Indigenous healthcare providers in PEOL care. The importance of an Indigenous Navigator also came forward in all focus groups. This was demonstrated in statements like:

*“They [the hospital] did have a navigator*,* and unfortunately*,* he’s leaving. I don’t know what happened*,* he hasn’t been here that long*,* that’s how bad it is to work in a hospital*,* it’s getting weary. It’s a shame that persons gone…I think it was too hard to work in the system. That’s a shame*,* because we need an interpreter when we get in that system.”* (Focus Group 2).

Advocacy to support First Nations People in dealing with grief also came forward in discussions. This was mentioned at the family and community level.

*“We just had a recent loss*,* two days ago. It’s constant. Sometimes in our community*,* there is a lot of grief*,* and a lot of grieving going on*,* and we recognize that but like I was saying*,* where I see gaps in care*,* is that we do a great*,* tremendous amount of support*,* prior and during*,* but after when everyone’s gone*,* family that have traveled here*,* they’re gone*,* and those supports are no longer required*,* those are gone…That’s the part that I would like to see in our community*,* more support for that*,* the ongoing grieving.”* (Focus Group 2).

(2) *Effective communication and shared decision making are vital between patients*,* families and healthcare providers.* Focus group participants felt that communication was vital between all parties involved in an individual’s PEOL care. This was described as necessary between healthcare providers, the patient and family, and the First Nations community’s healing centres.

*“We just went through that with my grandson at [the children’s hospital]. And they finally have a navigator there. But we were able to do the ceremonies that we needed to do. But we told them what we had to do*,* asked them what we needed*,* asked them if we could do this. We informed them right from the beginning right till the end.”* (Focus Group 1).

Participants also felt that improved communication at intake could help them respect important cultural practices and wishes during medical care. This was described in statements like:

*“I think when the person is very sick…I think that’s a good idea to have a form*,* or somewhere else to know when the family should be called*,* within what time frame*,* to respect if [they want a] sacred fire or to go to the synagogue…And then on this form*,* you could say*,* do you follow the traditional cedar bath? Do you want an elder from your community to come and speak to you? Or do you want us to call your community to get somebody to prepare the cedar bath? Like they would call here [the healing centre]. Have these questions on a form.”* (Focus Group 1).

(3) *Taking time to build trust between community members and healthcare providers*. The need to build trusting relationships between First Nations communities and healthcare providers to optimize services was evident within discussions. This came forth in statements like:

*“And that’s what the patient needs…they need somebody that will advocate on their behalf*,* because sometimes they speak but they’re not really heard*,* or they can’t get their point through as to what they want.”* (Focus Group 1).

*“There needs to be a study done on the lack of trust in medical profession because. [For example]*,* before you lift up a shirt to get in your stethoscope*,* ask them to hold that stethoscope. [A medical professional I know] mentioned that in class*,* and the professor looked at her and was like*,* ‘you don’t have time for that’. Yes*,* you do have time for that. It’s about working relationships*,* it’s about trust.”* (Focus Group 2).

Participants also felt it was beneficial for healthcare providers to be involved in their traditional ceremonies.

*“We had a ceremony in the healing room. It was really powerful…the attending nurse was there with her…even though she didn’t understand it*,* probably never been to anything [like that]*,* at any time in her life*,* but she felt it*,* because I was watching her. I was feeling it*,* and I was watching her to see if she was feeling the healing that was going on in that room right at that time.”* (Focus Group 2).

(4) *Cultural sensitivity training needed for healthcare providers and students*. When participants were asked about strategies which should be implemented to improve Western medical providers’ support of First Nations communities during PEOL care, cultural sensitivity training was consistently highlighted. This training was said to be needed at regular intervals for current healthcare providers, and during medical/healthcare providers training for trainees at an academic level.

*“Even if you went back to medical [school]*,* like you’re learning at school as doctors*,* there should be a component in there of respect. It doesn’t matter if it’s Indigenous or if it’s whoever…that should be part of the teaching…they barely touch on traditional medicines or even other medicines because they believe in Western medicine…That’s something that has to come together because it is going to happen whether the doctor wants it to.”* (Focus Group 2).

*“Cultural sensitivity and to adapt that to each community…We did that [cultural sensitivity training] years ago where we brought nurses and doctors into our community. So*,* that needs to start again*,* because over the years*,* staff have changed. And I know we’ve talked about it here a few times about doing that again*,* but it hasn’t been done. And the nurses*,* we can’t force them*,* but they should take the time to participate so that they understand First Nations people. There are nurses there and doctors that do want to learn. Because when I’ve been in the hospital*,* they’ve asked me questions and I said*,* “Well*,* wouldn’t it be nice if they had another session?…so cultural sensitivity training done on a continuous basis*,* not the one and done.”* (Focus Group 1).

Participants felt that cultural sensitivity training should be led by Indigenous community members who could provide detailed education using knowledge of their traditional medical practices.

*“And I think the culture sensitivity is really important*,* and it should be a mandatory course once you get hired…I think it’s important that they be taught by a Native person*,* because who knows it better than a Native person?…It was horrible when I was going to school and they were trying to teach us the Native culture*,* and it’s like*,* no. Or even trying to teach us the words. I’m like*,* ‘that’s not how you say that’. But of course*,* they’re right. They’re the teacher.”* (Focus Group 1).

Participants felt that this cultural sensitivity training, done in this way, would help to eliminate stigma, which they felt still existed in the Canadian healthcare system. This was evident in statements like:

*“First Nations people*,* we’re being not properly heard or treated.”* (Focus Group 1).

*“They didn’t treat him well when he first went in*,* because he had addiction problems. He wasn’t presently in addiction*,* but that’s all they saw*,* was that…he might be on meds*,* that he’s addicted*,* so they didn’t take any of his concerns [seriously].”* (Focus Group 2).

(5) * Create hospital policies and procedures that respect culture and traditional medical practices to support First Nations communities*. When discussing hospital policies and procedures that could be implemented to support First Nations communities, many participants felt these needed to be put into place to allow members of their community to die at home. This was discussed in statements like:

*“I think a while ago*,* we had a community member pass*,* and they passed in the hospital. And it was a little bit of an upset because the community member that passed really wanted to pass at home. So*,* I think it’s really important that the hospital doesn’t stop that. And they were waiting and waiting and waiting to get some kind of approval to take the community member back home. And I think that was cruel…So*,* that there*,* that’s something that needs to change.”* (Focus Group 1).

*“Hopefully a discharge program would be one thing we could probably look at. Discharge people from the hospital to home. Even those that are not dying*,* but they’re very*,* very sick. There’s lots of obstacles that the hospital wants them either to stay or we don’t have a bed space. That becomes an issue.”* (Focus Group 2).

Participants also stated that having sacred and traditional items available within the hospital would support their traditional practices and minimize financial burden on community members.

*“I think it would be beneficial to our community and our First Nation people*,* before they even leave to go to [a funeral home]*,* that the cedar bath is done because now [the funeral homes] are charging money for our people to go in there and do that bath. When my friend passed away 15 years ago*,* there was no fee for that…and now*,* they’re charging for that.”* (Focus Group 1).

*“[For the cedar bath] you have to use a white washcloth. And you start from the head and you go all the way around and wash. And then [you] take cloths…you don’t throw them in the garbage…As long as it’s a white cloth*,* it doesn’t matter where it’s from…it’s recommended to use a white [cloth]. I mean*,* we usually provide it here at the healing center and bring it to the ceremony. But it would be good if the hospital had it in the healing room and provided it so that we don’t have to worry about it.”* (Focus Group 1).

There was also concern that there were delays in releasing the ‘vessel’ (the body) post-death and participants stated the importance of timely release of the body.

*“When a person is passing*,* we want to look after them right away because we know their spirits like that vessel. We’re trying to send them…it’s a challenge.”* (Focus Group 2).

*“When my wife died in Sudbury…the next day after she passed that morning*,* in the afternoon*,* I called up [the funeral home] and everybody. Well*,* they [the hospital] can’t release the body for another couple days. ‘What?’ I said. I told [the funeral director] ‘I’m going to go up there and get her. I’m just going to put my back seat down and I’ll put her back there and bring her to you.’…They were going to keep her and that would interfere with our [practices]. That’s when I…thought about the Western medicine…My concern is about that*,* when bodies are being held*,* longer than they should be.”* (Focus Group 2).

Table 3 lists traditional medical practices mentioned by participants in our focus groups.

**Table 3:** Traditional medical practices occurring during palliative or end-of-life care as described by participants:

Gathering around the dying persons and their families for support*
Sacred fire, lit after someone passes*
Cedar baths for healing, stress relief and transition*
Final feasts, memorial gatherings, and send-off ceremonies*
Final goodbyes with traditional prayers and thanksgiving
Singing, drumming, sweat lodge ceremonies
Prayers and herbal medicines for balance/healing
Provide protection and assistance to the spirit of the deceased person
Directional traditions during ceremonies (e.g. carrying the coffin in specific paths)
Unique forms of grieving: sharing stories and humour
Connection of spirits and the role of ceremonies in guiding them
Sacred objects (tobacco, pipe, cedar water/leaves, sage, white washcloths)
Special roles (fire watchers, deceased person’s body watchers, and feather carriers)

*Mentioned in all three focus groups

Participants stated that hospital policies and procedures needed to allow these traditional medical practices within the hospital.

*“It’s happening more and more now where people are asking for that cedar bath when someone passes. It’s usually the family*,* and it’s usually four…women doing it*,* but not necessarily. I’ve done a few of them myself because there’s no one available…and you’re not allowed to cry. That’s what I was told*,* you weren’t allowed to cry in the cedar bath.”* (Focus Group 1).

*“It’s not just the cigarettes*,* singing*,* the ceremony*,* even simple acts*,* not just smelling that medicine*,* it could be the sage*,* or it could be the sweet grapes*,* or it could be the cedar*,* and even just giving them water and berries*,* stuff like that. We talk about how important strawberry is.”* (Focus Group 2).

Within the focus groups, the importance of hospitals creating locations (i.e., healing/culture rooms) for ceremonies and traditions to be held was clearly described. Within these locations, participants felt it was important to minimize visitor restrictions, ensure the space is peaceful, and have spaces for gathering of family and community members.

*“Because he was there in his palliative care unit…he’s going to die*,* and that’s the focus. When we came*,* the family came*,* and we shared stories. He was bright. The guitarist sang*,* so I don’t know if that would be able to happen at this palliative care room. We have to have more than one person*,* or two people in a room. It can’t be just one because…it’s a big family.”* (Focus Group 2).

*“Having a location that’s big enough for the individual who is very sick to share a meal with their family*,* anyone in the larger community if they want to is really important.”* (Focus Group 1).

## Discussion

In Northern Ontario, where a higher percentage of the population identifies as Indigenous compared to other parts of the province, there is a strong need for culturally sensitive healthcare that respects Indigenous traditions and knowledge. Supporting Indigenous learners is critical to developing healthcare providers who understand traditional practices and can deliver culturally appropriate care.

Within Northern Ontario, more than 17% of the population identify as Indigenous, compared to less than 5% in other regions of the province [3]. This study sought to address these gaps by working alongside First Nations communities. Through community-driven engagement, it aimed to explore how Indigenous individuals and families define a “good death,” including the roles of family, community, and healthcare providers in PEOL care, and propose actionable recommendations for culturally sensitive practices. By centring Indigenous voices and traditions, this research project hopes to facilitate policy and healthcare practices that honour the values and needs of First Nations communities, ensuring equitable and meaningful PEOL care for all.

The findings of this study highlight important gaps in the healthcare system’s ability to meet the Calls to Action from Canada’s Truth and Reconciliation Commission, (specifically actions talking about health, 18–24) [3]. The data, gathered through sharing sessions, emphasize the importance of family, community, and cultural practices in defining what a “good death” is for First Nations communities. The preference for dying at home and the central role of family caregivers reflect cultural values that emphasize the significance of family and community connections. These insights are pivotal for healthcare providers, who should incorporate cultural sensitivity into PEOL care by building trust, supporting home-based care, and respecting spiritual beliefs. Collaboration with community leaders and caregivers is also key to bridging the gap between Western healthcare systems and the cultural sensitivity needs of First Nations communities. Although the study benefits from strong community involvement and valuable qualitative data, its small sample size and limited geographic scope may reduce the ability to apply the findings broadly. Sharing the results with local healthcare providers is an important step in integrating culturally appropriate care into current PEOL care practices.

Our findings also underscore key system-level implications. Participants repeatedly emphasized the value of Indigenous Navigators in facilitating communication, advocating for patient and family needs, and creating culturally safe spaces. We recommend that hospitals serving Indigenous communities invest in full-time Indigenous Navigator roles as essential positions within hospitals and liaisons person for palliative care teams. Additionally, participants described the spiritual importance of traditional rituals (such as cedar baths, sacred fires, and final feasts), particularly during hospitalization. We recommend that institutions consider designating ceremonial or healing spaces that allow families to conduct such practices in a respectful and private setting. These initiatives reflect practical steps toward fulfilling the Calls to Action 22 and 23, and honouring Indigenous ways of knowing and caring [3]. The historical context of colonization, residential schools, and systemic racism continues to shape healthcare experiences and trust for First Nations peoples. The legacy of forced assimilation disrupted end-of-life ceremonies, spiritual care, and intergenerational caregiving. These histories remain embedded in how communities define a “good death,” and should be central to understanding gaps in access, communication, and comfort with Western care systems.

These findings provide a strong foundation for improving PEOL care in the participating communities. This study shows how partnerships between Indigenous communities and Western healthcare providers can lead to culturally safe and sustainable care. By focusing on the identified themes, healthcare providers can better respect and honour Indigenous traditions and values, ensuring care that aligns with the cultural understanding of a “good death.” As researchers embedded in these communities, our goal was not just to document culturally defined PEOL care, but also to act on it. The findings from this study will be used to inform culturally grounded provider education, strengthen partnerships with local First Nations communities in Canada, and support hospital policy development that allows for culturally safe practices.

### Limitations

While this study included three First Nation communities in rural northern Ontario, the findings are limited to those communities. Additionally, Indigenous community members were not involved in coding the data. However, they did take part in reviewing the findings and feedback, which helped shape our interpretations. Each First Nation community is unique and while they may share many traditional practices, their PEOL practices may not be the same as those discussed within this paper. This highlights the need for continued collaboration throughout Canada to ensure all Canadians can receive appropriate, culturally safe end of life care.

## Conclusion

This study demonstrates that a “good death” is culturally defined and deeply rooted in family, community, and spiritual beliefs. Culturally sensitive PEOL care must honour these elements to improve care experiences for First Nations communities. In response to the findings, we will use this knowledge to enhance local healthcare education, inform policy development, and promote culturally appropriate hospital practices (such as access to Indigenous Navigators and ceremonial accommodations). These actions support an evolving model of care that is responsive, respectful, and rooted in cultural safety.

## Data Availability

Access to study data can be requested by contacting the corresponding author.
